# Stem Cells and Irradiation

**DOI:** 10.3390/cells10040760

**Published:** 2021-03-30

**Authors:** Alain Chapel

**Affiliations:** 1 Service de Recherche en Radiobiologie et en Médecine Régénérative (SERAMED), Laboratoire de Radiobiologie des Expositions Médicales (LRMED), Institut de Radioprotection et de Sûreté Nucléaire (IRSN), F-92260 Fontenay-aux-Roses, France; alain.chapel@irsn.fr; Tel.: +33-1-5835-9546; 2 Centre de Recherche Saint-Antoine, CRSA, INSERM, Sorbonne Université, Saint Antoine Hospital, 75012 Paris, France

The main difficulty of radiotherapy is to destroy cancer cells without depletion of healthy tissue. Stem cells and cancers are tightly interrelated. On the one hand, radiosensitivity/radioresistance of cancer stem cells affects the radiocurability of tumors, on the other hand, radiosensitivity is responsible for the stem cell depletion of organs at risk exposed to irradiation. Efficient solid cancer destruction is limited by the preservation of organ homeostasis. For this reason, targeted irradiation is an effective cancer therapy, however, damage inflicted to normal tissues surrounding the tumor may cause severe complications. The consequences of stem cell depletion of healthy tissue irradiated are acute and chronic radiation diseases. The depletion of endogenous stem cells can be compensated by a supply of exogenous stem cells. For this reason, cell therapy is a therapeutic approach that offers a therapeutic alternative to patients who have failed conventional treatment. This domain will bring forth the solution for optimal radiocurability associated with long-term patients’ quality of life.

This special issue covers research on the radiosensitivity of cancer stem cells and adult stem cells associated with tissue regenerative medicine.

Integration of the cross talk of these two types of stem cells is essential. Nagle and colleague studied the roles of organoids as model to understand relationship between normal tissue and tumor responses in radiobiological studies [1]. Understanding the radioresistance mechanisms of cancer cells is fundamental in order to be able to eradicate the tumor while preserving healthy tissue. Two research articles dealt with cancer cells. The article of Park and colleagues demonstrated that SOX2 contributed to the induction of colorectal cancer cells and is regulated by radio-induced activation of the PI3K/AKT pathway [2]. Kamble and colleagues studied the relationships between radioresistance and breast cancer stem cells mediated by Nrf2-Keap1 pathway. Nrf2-Keap1 signaling controls mesenchymal–epithelial plasticity and regulates tumor-initiating ability and promotes the radioresistance of breast cancer stem cells [3].

The consequence of restoring the homeostasis of healthy tissue is first of all to allow tissue regeneration and then organ functionality on a permanent basis. A supply of mesenchymal stromal cells (MSCs) ensures this functionality, mainly through a trophic effect. 

Two research articles dealt with MSCs in the treatment of radiological burns. Brunchukov and colleagues studied the effect of human MSCs derived from the placenta and their conditioned medium concentrate on skin-regenerative processes. The use of conditioned MSCs in severe local radiation injuries accelerates the transition of the healing process to the stage of regeneration and epithelization [4]. Cavallero and colleagues settled a method founded on an in vitro bioengineering of human skin organoids, joined with in vivo xenografting in immune-deficient mice. This model was used to understand significances of exposure epidermal stem cells to low-dose irradiation and their consequences in radio-dermatitis [5]. Thanks to its trophic effect, the treatment of healthy tissue by MSCs seems to be able to address many of the pathologies resulting from radiotherapy. Synthesis reports explore the recent progress and discuss the future perspectives about MSCs and MSC-exosomes for mitigating radiotherapy side effects [6,7,8].

Each pathology related to radiotherapy is complex, which is why it is necessary to apprehend all the elements of a pathology and its treatment but also to be able to understand in detail the associated mechanisms. Helissey and colleagues reviewed radiation cystitis and its treatments. The authors investigated the role of immunity with a special focus on macrophages. They concluded that MSCs seem to be an excellent therapeutic substitute for the treatment of fibrosis in chronic radiation cystitis [9].

To go further into the abovementioned issue and end on an optimistic note, it is interesting to associate the beneficial effect of irradiation with cell therapy in order to propose novel treatments for new pathologies. Tovar and colleagues tried a therapeutic approach, based on MSCs stimulated with radiation, to improve pneumonia caused by SARS-CoV-2. The activation of the immune system by the irradiated tumor to trigger the beneficial abscopal effect is decisively improving radiotherapy applications and their outcomes [10]. 
